# Supplementary material for: Ratoon Rice Cropping Mitigates the Greenhouse Effect by Reducing CH4 Emissions through Reduction of Biomass during the Ratoon Season
Source: Plants (Basel). 2023 Sep 22;12(19):3354. doi: 10.3390/plants12193354 (PMC10574029; doi:10.3390/plants12193354)
Supplement: Supplementary file 1 [file plants-12-03354-s001.zip › plants-2558580-supplementary.pdf]

Supplementary Materials

Ratoon Rice Cropping Mitigates Greenhouse Effect by Reducing CH_4 Emissions through Reduction of Biomass during the Ratoon Season

Xiaojian Ren [1], Kehui Cui [1,*], Zhiming Deng [1], Kaiyan Han [1], Yuxuan Peng [1], Jiyong Zhou [2], Zhongbing Zhai [2], Jianliang Huang [1] and Shaobing Peng [1]

1 National Key Laboratory of Crop Genetic Improvement, Key Laboratory of Corp Ecophysiology and Farming System in the Middle Reaches of the Yangtze River, Ministry of Agriculture and Rural Affairs, College of Plant Science and Technology of Huazhong Agricultural University, Wuhan 430070, China

2 Wuxue Agro-Technology Extension Service Center, Wuxue 435499, China
* Correspondence: cuikehui@mail.hzau.edu.cn

Table S1 Unit prices of agricultural inputs and estimated outputs.

Item	Unit price	Unit	Reference
Nitrogen fertilizer (urea, N content= 46%)	3.80	CNY kg^{-1}	[53]
Phosphate fertilizer (superphosphate, P_2O_5 content= 12%)	1.25	CNY kg^{-1}	[25]
Potassium fertilizer (potassium chloride, K_2O content= 60%)	2.00	CNY kg^{-1}	[53]
Herbicides	175.00	CNY kg^{-1}	[53]
Insecticides	100.00	CNY kg^{-1}	[53]
Fungicides	90.00	CNY kg^{-1}	[53]
Rice seed (Indica)	55.00	CNY kg^{-1}	[53]
Labor	83.00	CNY per person per day	[24]
Carbon-trade price	232.70	CNY t^{-1} CO_2-eq	[24]
Rice grain of the first season (Indica)	2.06	CNY kg^{-1}	[54]
Rice grain of the second season (Indica)	2.24	CNY kg^{-1}	[54]

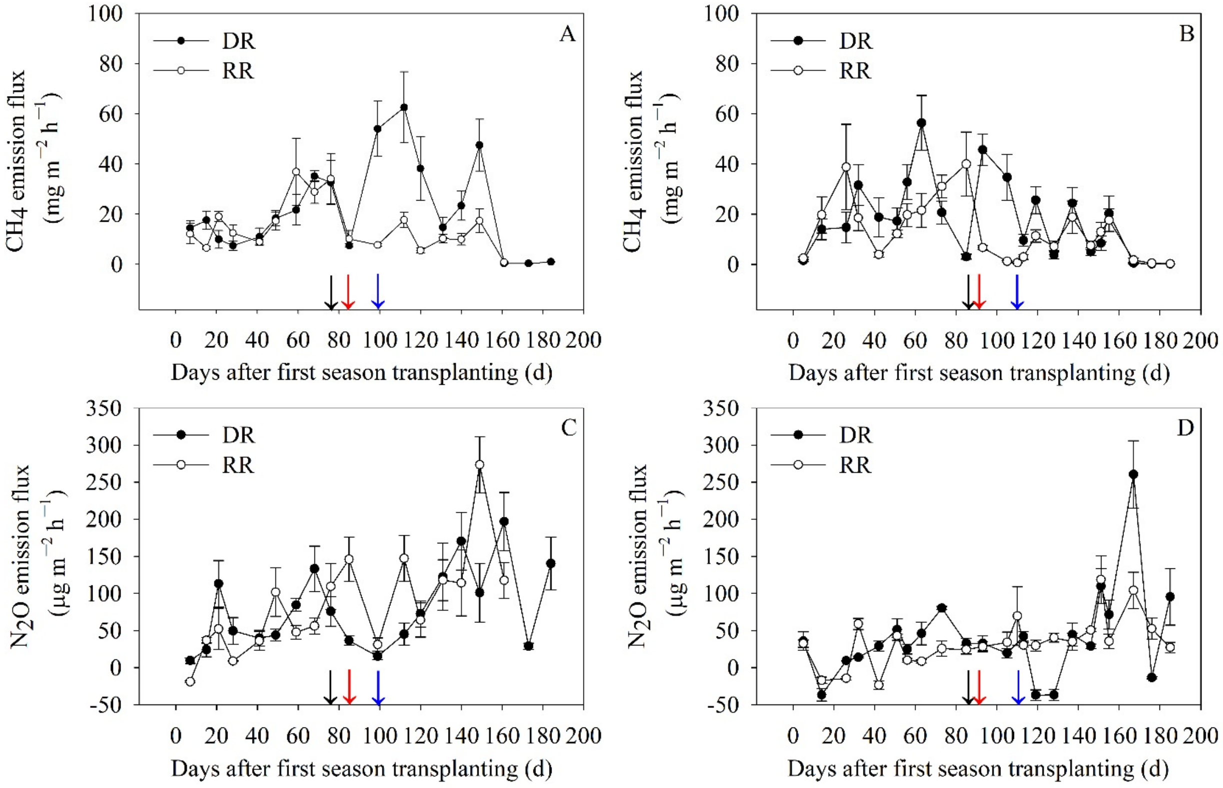

Figure S1 CH_4 emission flux in 2018 (A) and 2019 (B) and N_2O emission flux of DR and RR in 2018 (C) and 2019 (D) in double rice cropping system (DR) and ratoon rice cropping system (RR).

The first season refers to early season of DR and main season of RR, and the second season refers to late season of DR and ratoon season of RR. Black arrow indicates the harvest date of the early season rice, red arrow indicates the transplanting date of the late season rice, and blue arrow indicates the harvest date of harvest of the main season.

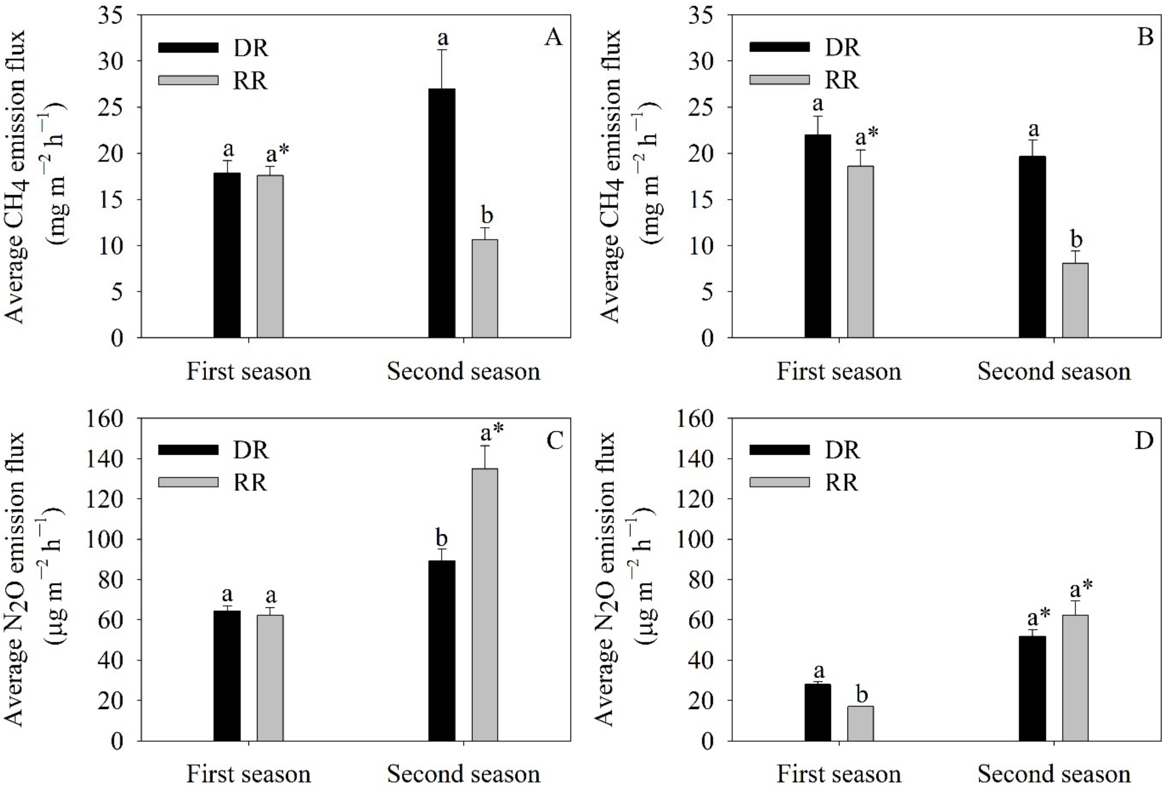

Figure S2 Seasonal average CH_4 emission flux in 2018 (A) and 2019 (B) and seasonal average N_2O emission flux in 2018 (C) and 2019 (D) in double rice cropping system (DR) and ratoon rice cropping system (RR).

The first season refers to early season of DR and main season of RR, and the second season refers to late season of DR and ratoon season of RR. Different lowercase letters above the bars indicate significant differences ($P < 0.05$) between cropping systems in the same season. * indicates significant differences ($P < 0.05$) between the two seasons in the same cropping system.

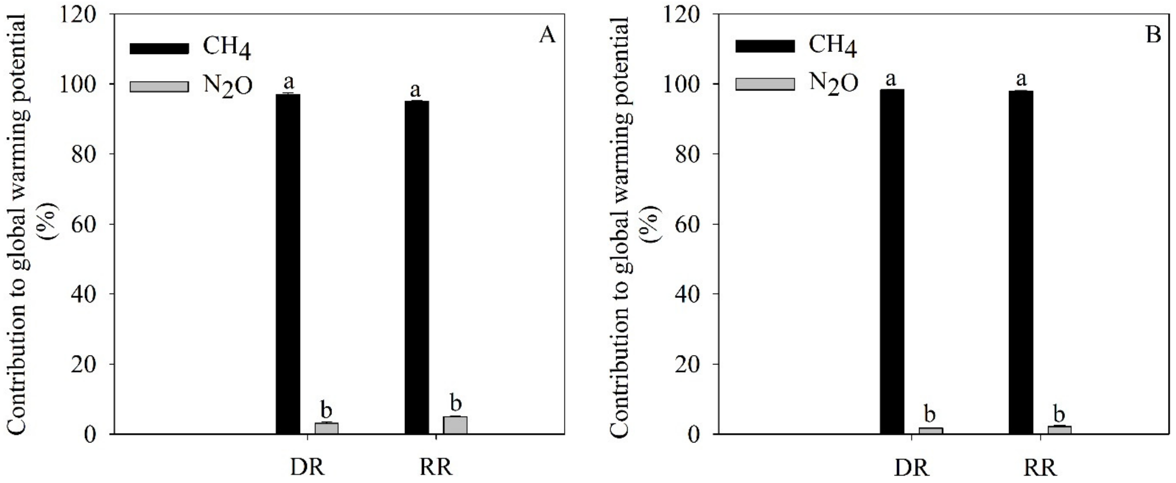

Figure S3 Contribution of CH_4 and N_2O to greenhouse warming potential (GWP) in double rice cropping system (DR) and ratoon rice cropping system (RR) in 2018 (A) and 2019 (B).

Different lowercase letters above the bars indicate significant differences ($P < 0.05$) between the two gases.

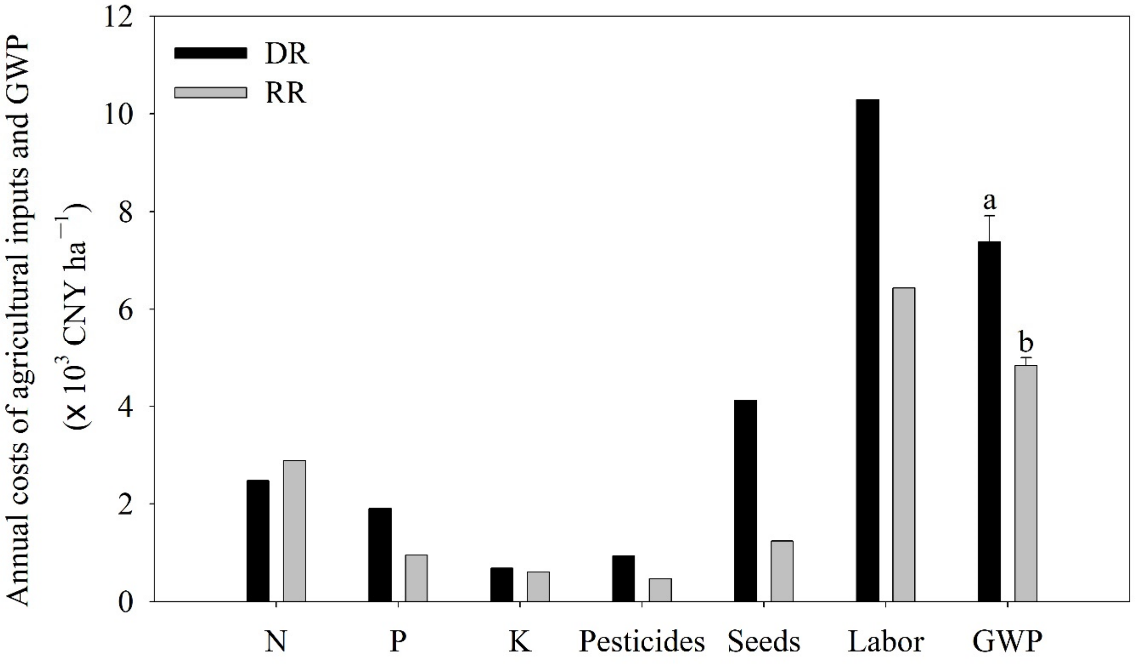

Figure S4 Annual costs of agricultural inputs and greenhouse warming potential (GWP) in double rice cropping system (DR) and ratoon rice cropping system (RR).

Different lowercase letters above the bars indicate significant differences ($P < 0.05$) between the two cropping systems.

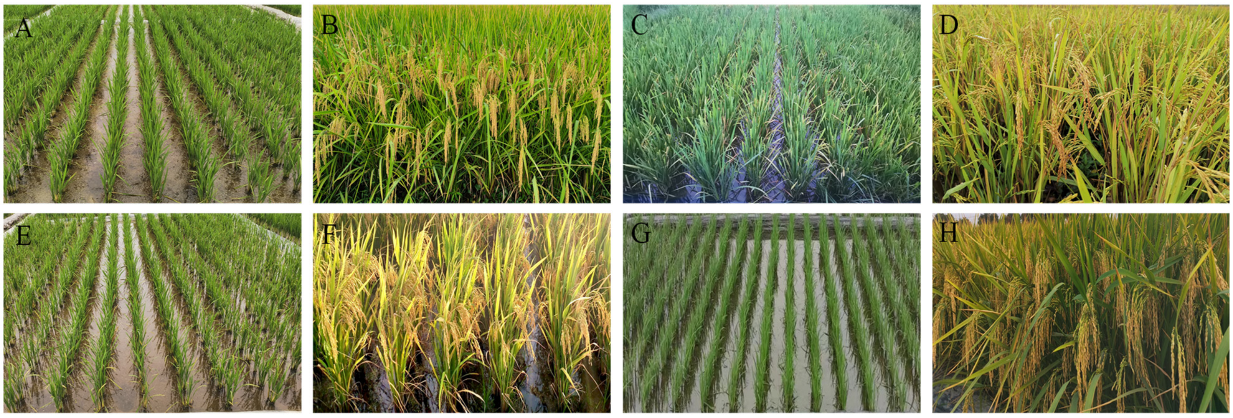

Figure S5 Rice plants at the tillering and maturity stages in the two seasons in double rice cropping system (DR) and ratoon rice cropping system (RR). (A) Rice plants at tillering stage in the main season in RR. (B) Rice plants at maturity stage in the main season in RR (provided by Yang Chen). (C) Regenerated rice plants after harvest of the main crops in RR. (D) Rice plants at maturity stage in the ratoon season in RR. (E) Rice plants at tillering stage in the early rice. (F) Rice plants at maturity stage in the early rice (provided by Huang Jianda). (G) Rice plants at tillering stage in the late rice. (H) Rice plants at maturity stage in the late rice.
